# Supplementary material for: UNC-45 assisted myosin folding depends on a conserved FX3HY motif implicated in Freeman Sheldon Syndrome
Source: Nat Commun. 2024 Jul 25;15:6272. doi: 10.1038/s41467-024-50442-6 (PMC11272940; doi:10.1038/s41467-024-50442-6)
Supplement: Supplementary file 1 — Supplementary Information [file 41467_2024_50442_MOESM1_ESM.pdf]

Supplementary Figures

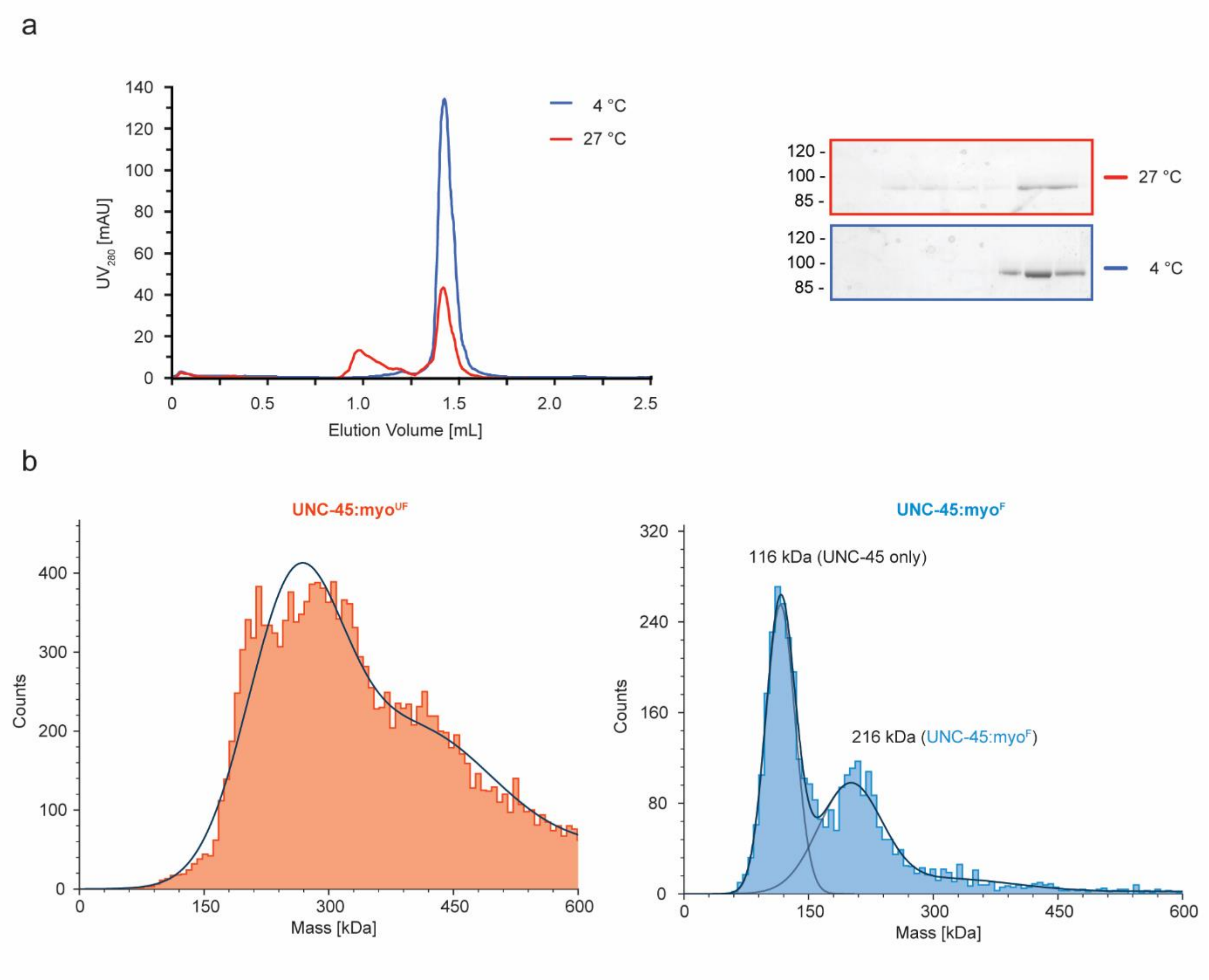

Supplementary Figure 1: Characterization of the UNC-45:Myo^{UF} and UNC-45:Myo^{F} complexes

(a) SEC of recombinant myosin incubated at 4°C (folded) and at 27°C (partially unfolded). SDS-PAGE of the peaks is shown on the right. **(b)** Mass photometry experiments of UNC-45:Myo^{UF} and UNC-45:Myo^{F} complexes.

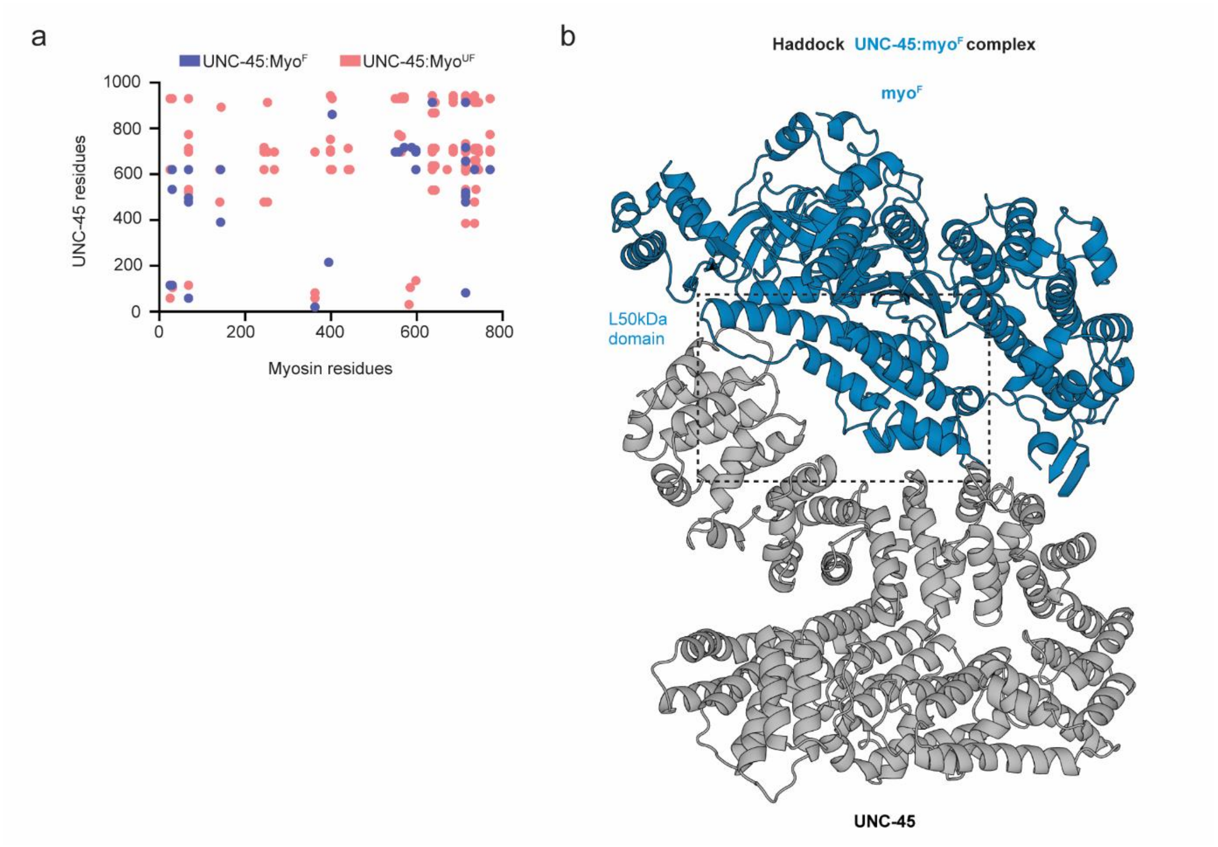

Supplementary Figure 2: UNC-45:Myo^{F} and UNC-45:Myo^{UF} show distinct crosslink patterns compatible with folded and unfolded myosin.

(a) Plot of interlinks comparing complexes with UNC-45 containing intact (blue) or damaged (red) myosin. Links have been filtered to FDR ≤ 5%. **(b)** Model of the UNC-45:Myo^{F} generated using Haddock and the cross-linking restraints. The lower 50kDa domain of myosin points at the UCS domain of UNC-45.

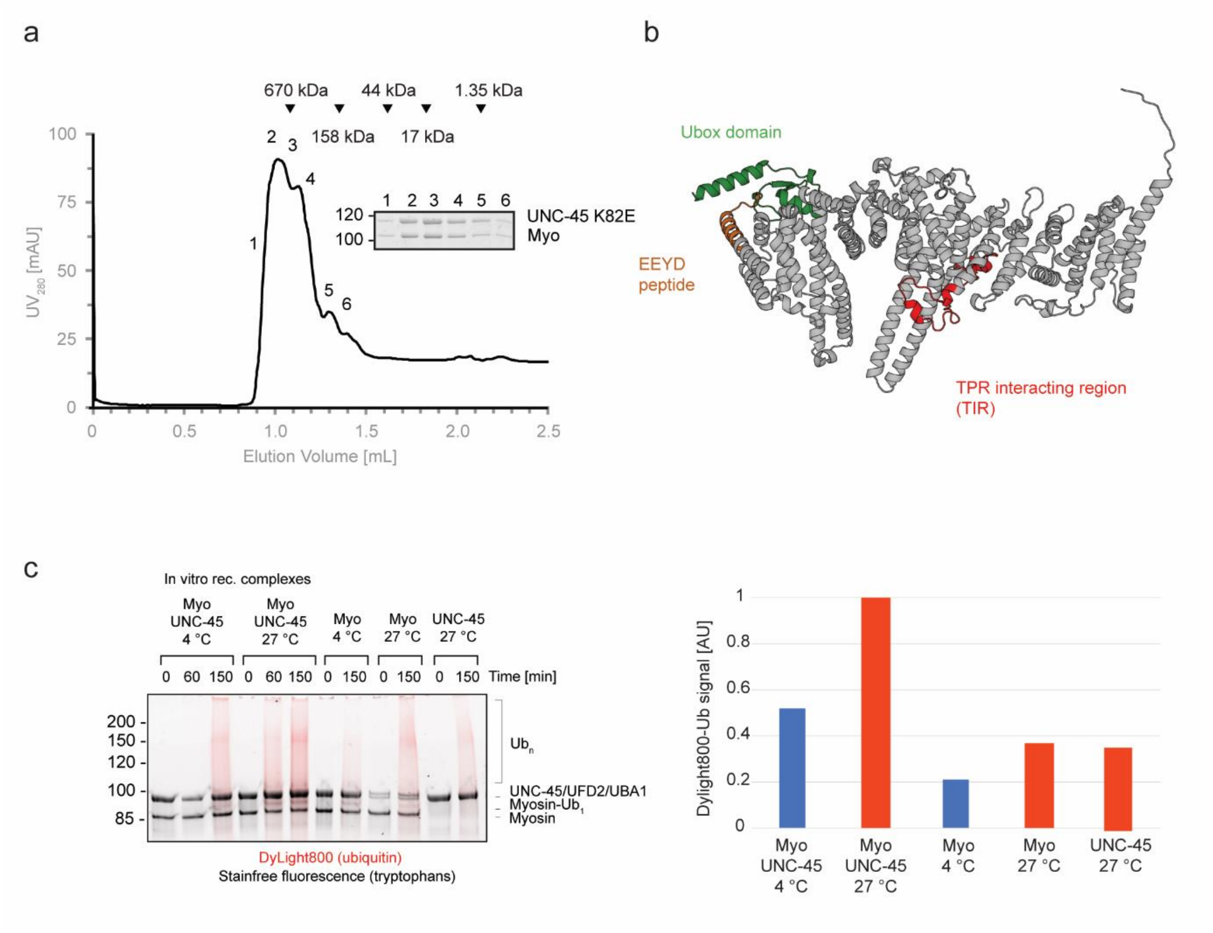

Supplementary Figure 3: UFD-2 specifically targets damaged myosin for ubiquitination.

(a) Purification of the UNC-45:myosin complexes in the UNC-45 K82E background. **(b)** AlphaFold2 model of UFD2 with Ubox (green) and the TPR-interacting region (red) identified in Fig. 2c. The peptide containing the internal EEYD is labeled in orange.**(c)** Left: UFD-2-mediated ubiquitination of *in vitro* reconstituted UNC-45:myosin complexes and UNC-45 or myosin alone performed at 4 °C and 27 °C. SDS-PAGE gels with signals of stain-free fluorescence (total protein) and DyLight800 (ubiquitin) have been overlayed. Right: Quantification of Ub_n after 150 minutes using DyLight800 signal.

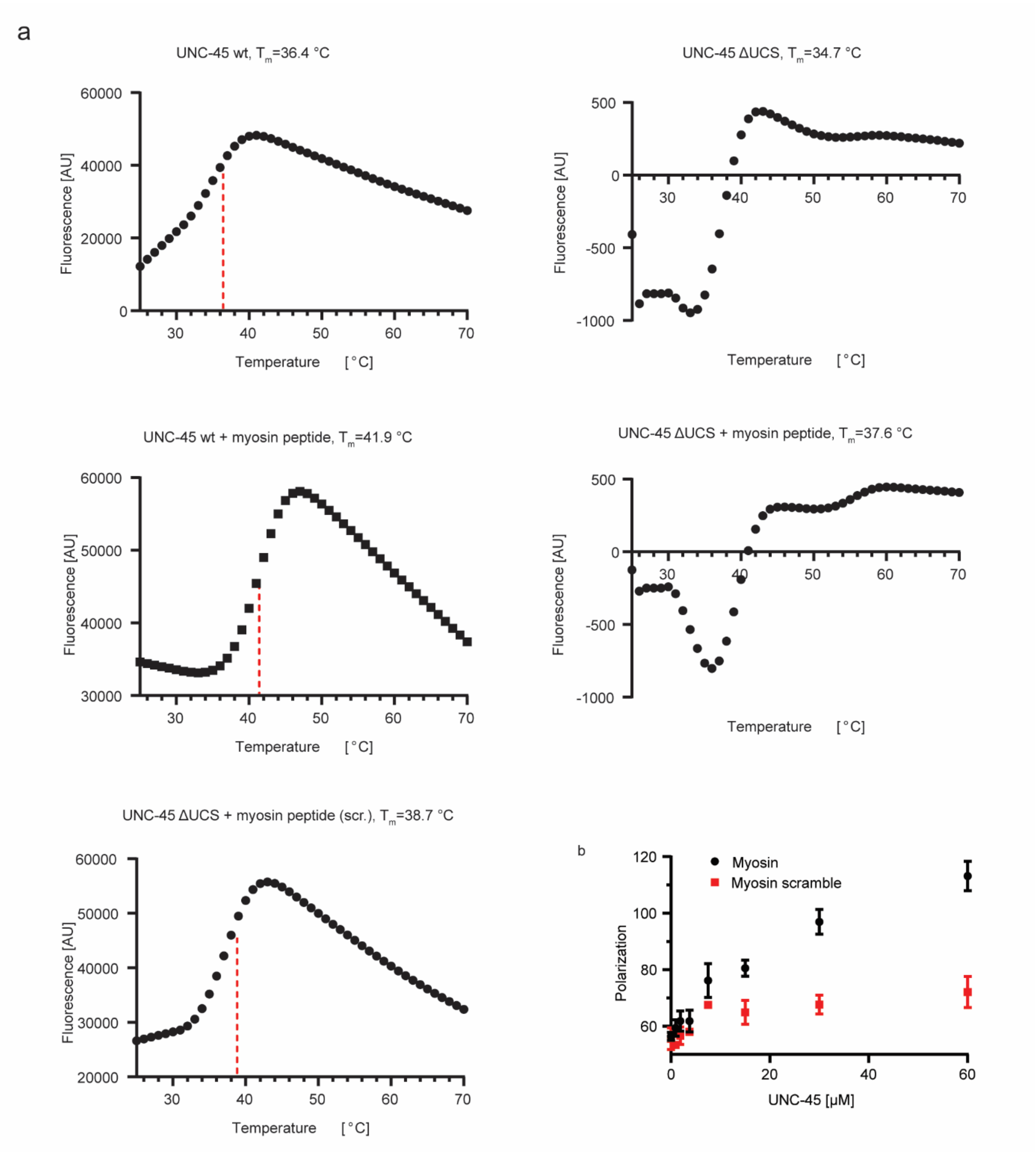

Supplementary Figure 4: A discrete myosin peptide specifically binds to the UNC-45 UCS domain.

(a) Selected, representative melting curves of thermal shift assays. Red dashed lines indicate the determined melting temperatures (T_m). **(b)** Fluorescence anisotropy of myosin peptides. For measurements the minimal motif shown in figure **Fig. 3b** was used. Mean and standard deviation of 3 replicates (WT) and 2 replicates (scrambled) are shown.

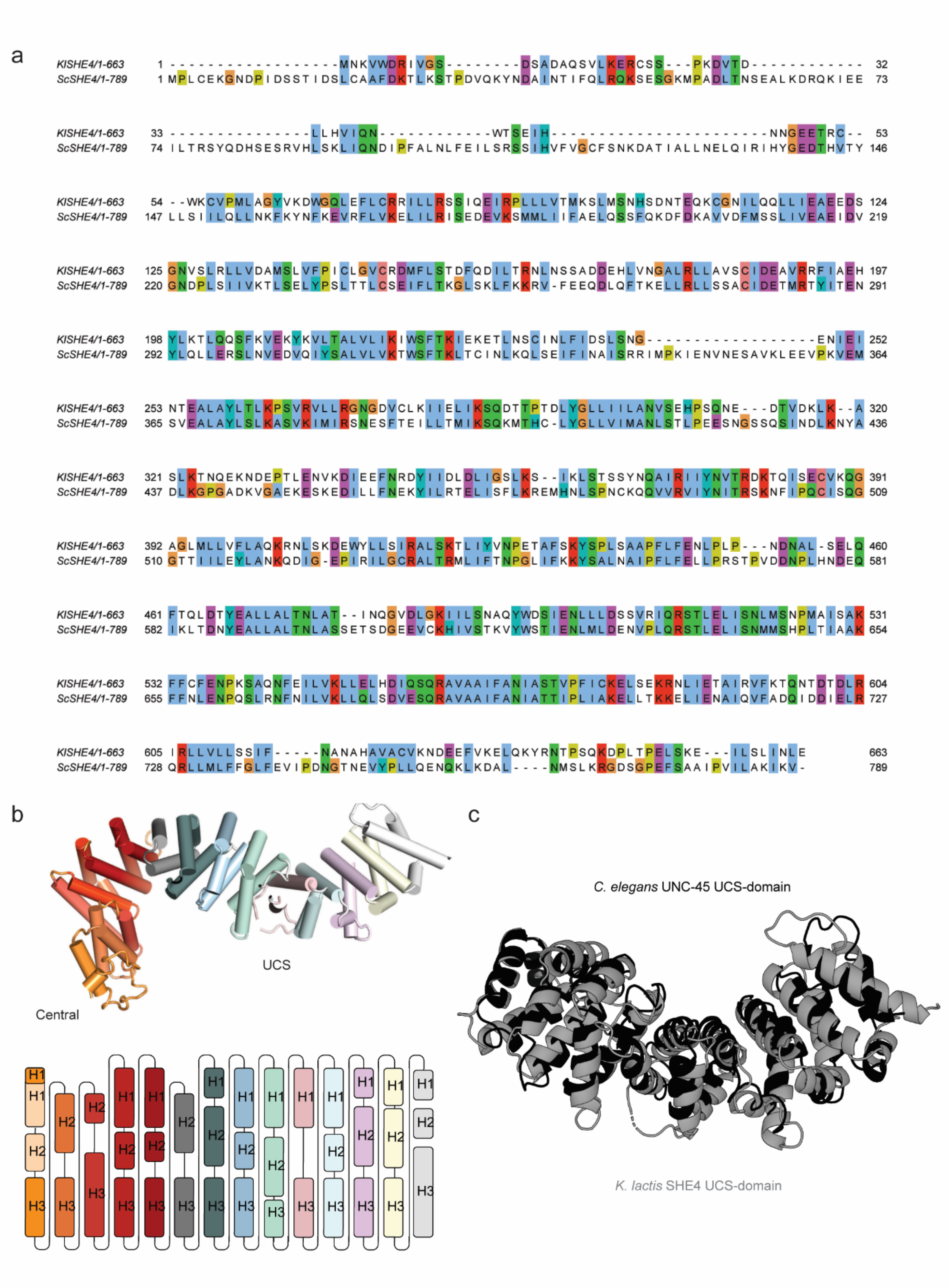

Supplementary Figure 5: *K. lactis* SHE4 crystal structure.

(a) Alignment of SHE4 comparing the sequences from the *K. lactis* chaperone used in this study and *S. cerevisiae* that has been described previously in Shi & Blobel, 2010.

(b) *K. lactis* SHE4 structure at 2.4 Å in absence of a myosin peptide, with α-helices shown as cylinders to indicate the ARM-repeats (top), and schematic representation of ARM-repeats topology (bottom). **(c)** Superposition of *K. lactis* SHE4 (grey) and *C. elegans* UNC-45 (PDB: 4i2z, black) UCS domains, that align well to each other (root mean square deviation of 3.8 Å over 321 Cα-atoms).

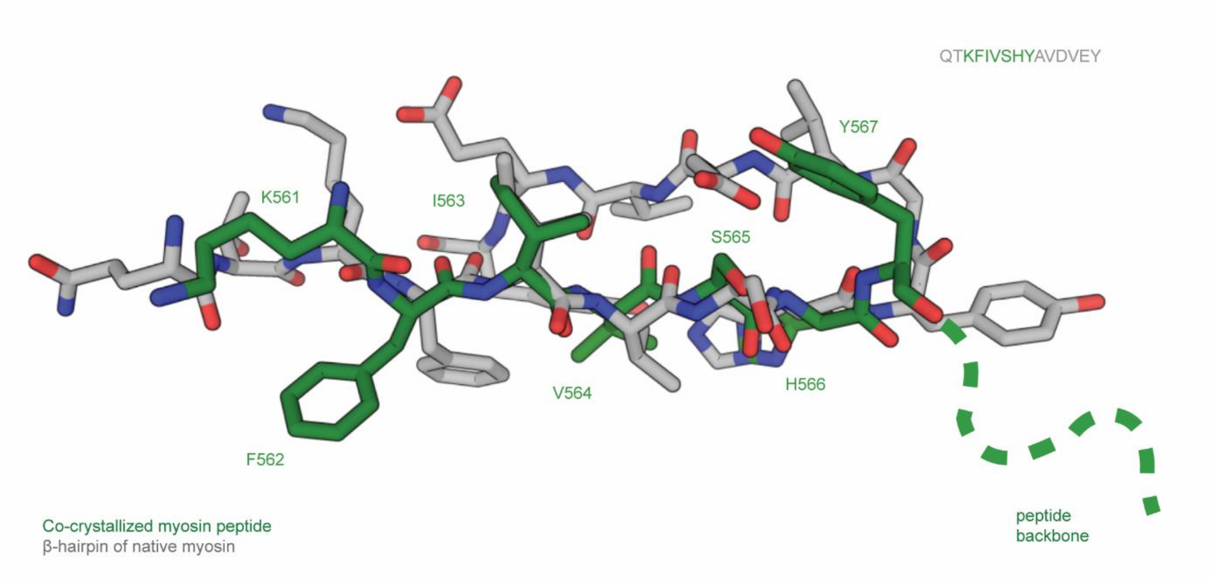

Supplementary Figure 6: *K. lactis* SHE4 binds the myosin peptide in an extended conformation.

Structural alignment of the co-crystallized myosin peptide (green) and the native myosin β-hairpin loop of myo4 (grey), indicating the different backbone conformations free (β-hairpin) and chaperone-bound (extended) myosin motif.

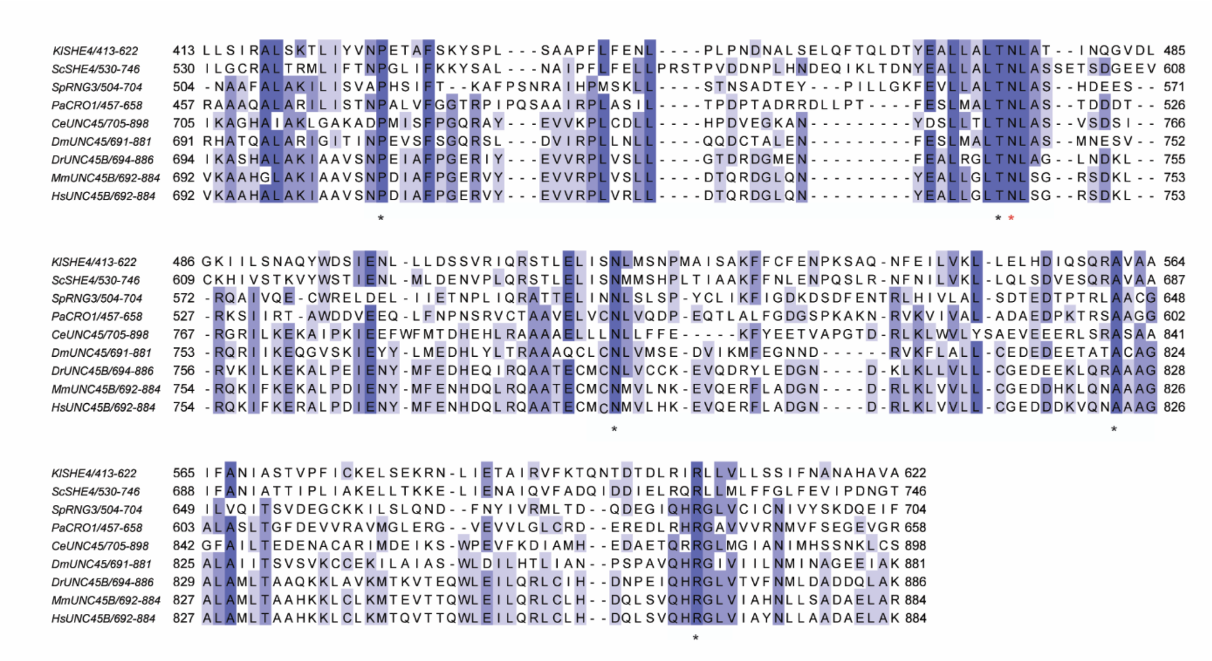

Supplementary Figure 7: *K. lactis* SHE4 binds the myosin peptide via a conserved interface.

Sequence alignment of the myosin binding region of UCS-proteins from selected species. Strictly conserved residues that are involved in myosin binding are highlighted with a black asterisk, Asn475 mutated in Fig. 4c is highlighted with a red asterisk. Residues are coloured in shades of blue according to percentage identity with dark blue >80% and white <40%.

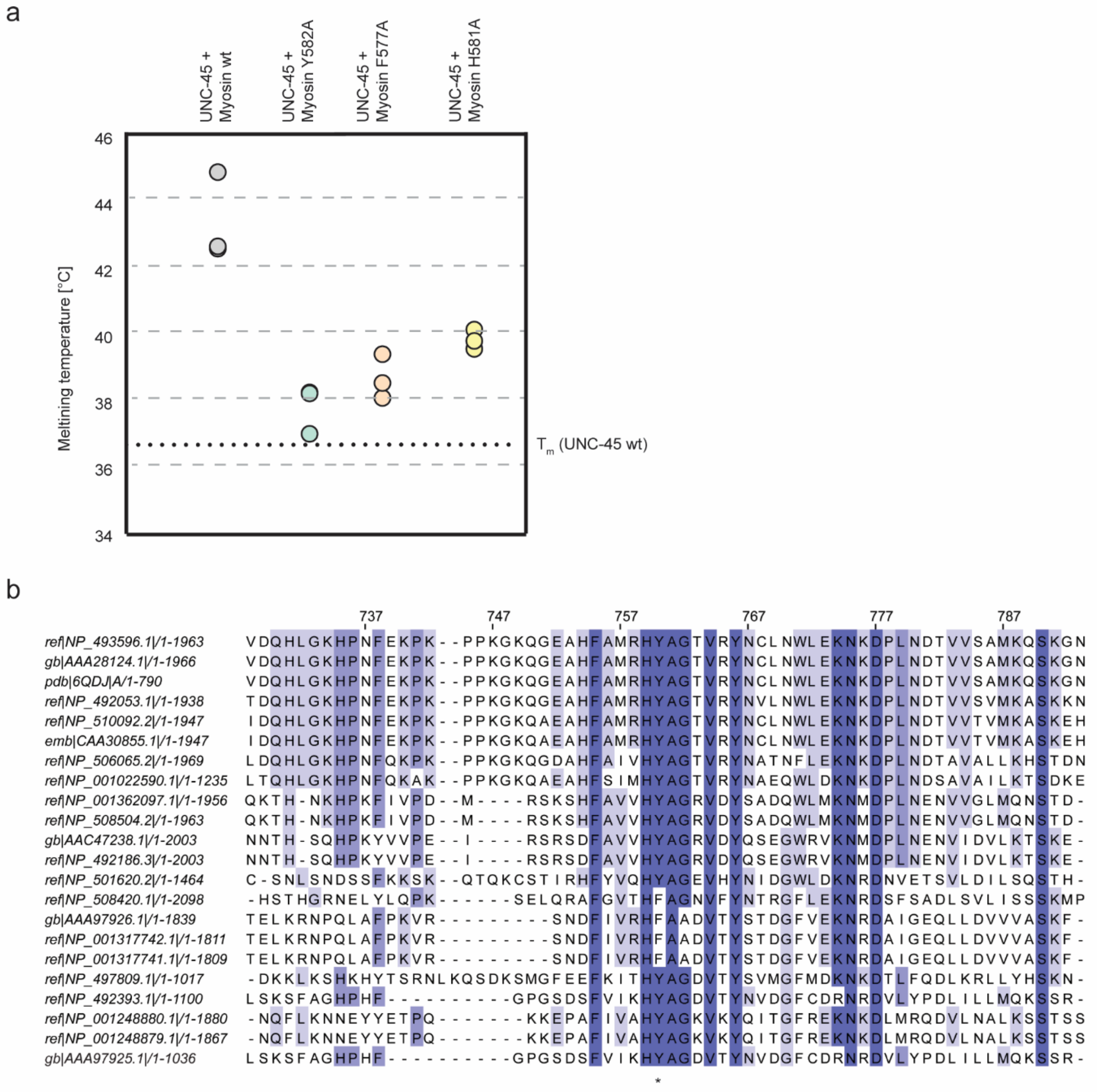

Supplementary Figure 8: Validation of the FX$_3$HY-motif for UNC-45 binding and conservation among *C. elegans* myosin isoforms.

(a) Thermal shift assays comparing melting temperatures of UNC-45 in presence of the WT myosin peptide versus different mutants of the FX$_3$HY-motif. Dotted line indicates the melting temperature of WT UNC-45 in absence of peptides as observed in **Fig. 3c**. **(b)** Conservation of FX3HY-motif in *C. elegans* myosin isoforms. Position 582 is indicated with a red asterisk, displaying either Y or F.

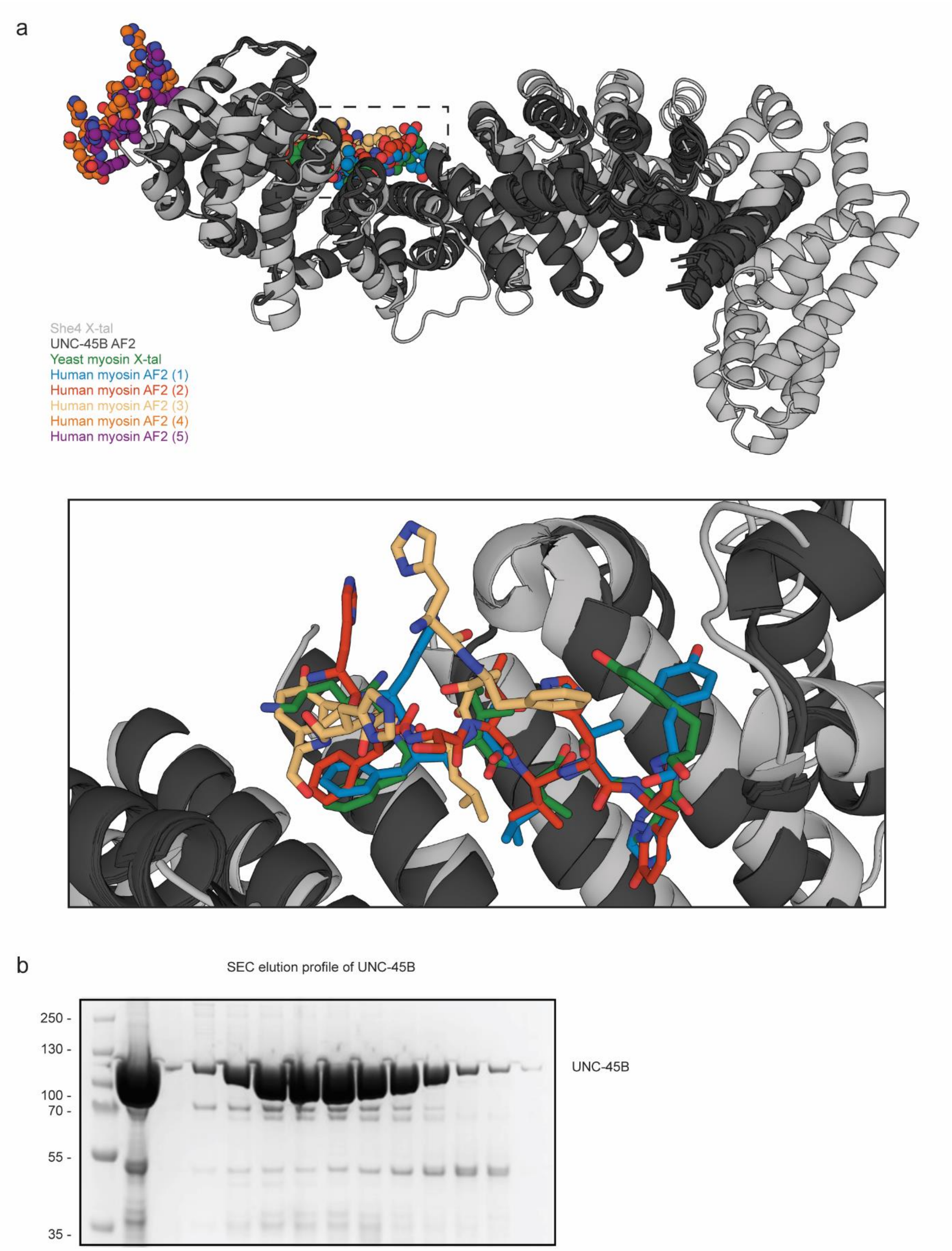

Supplementary Figure 9: Binding of MYH3 peptide to human UNC-45B

(a) Top: Alphafold2 models of UNC-45B (dark grey) in complex with MYH3 peptides (blue rank1, red rank2, yellow rank3, orange rank4, purple rank5), superposed to She4 (light grey) and the myosin peptide (green) of the crystal structure determined in this

paper. Only the UCS domain (aa 452-931) and the minimal MYH3 peptide HFSLIHY are shown. Bottom: Zoom-in showing modelled peptides (blue, red, yellow) together with the co-crystallized FX3HY peptide (green) bound to SHE4. **(b)** SDS-PAGE analysis of isolated UNC45B after SEC purification, showing purity of protein sample.

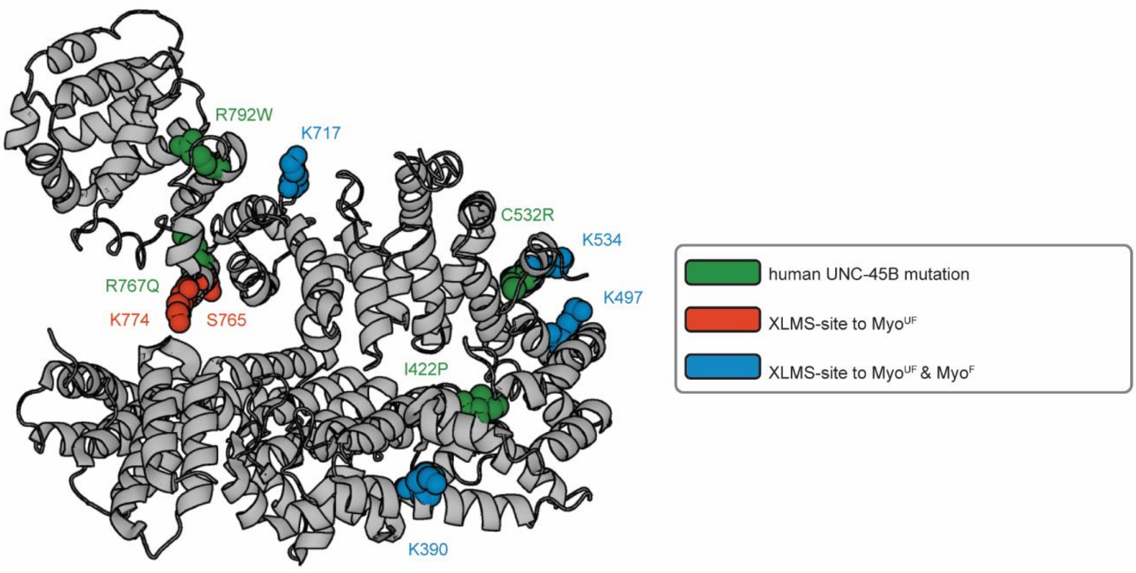

Supplementary Figure 10: Comparison of crosslink sites to myosin and human point mutation causing myopathies mapped on the UNC-45 structure.

Mapping of residues on the *C. elegans* UNC-45 structure (PDB: 4i2z) of which point mutations have been linked to human myopathies (blue). Magenta and green indicate interlinks found in UNC-45:myo^{F} only or both UNC-45:myo^{UF} and UNC-45:myo^{F}.

Supplementary Table

Supplementary Table 1 – Data collection and refinement statistics

	She4_apo	She4_pep
PDB ID	8BRG	8BRH
Wavelength (Å)	0.9763	1.1512
Resolution range	19.83 - 2.4 (2.49 - 2.4)	49.47 - 2.4 (2.49 - 2.4)
Space group	P 31 2 1	P 31 2 1
Unit cell	129.51 129.51 77.19 90 90 120	128.48 128.48 77.54 90 90 120
Total reflections	581084 (59265)	285131 (28464)
Unique reflections	29424 (2908)	34958 (3457)
Multiplicity	19.7 (20.4)	8.1 (8.1)
Completeness (%)	99.74 (99.97)	99.92 (99.86)
Mean I/sigma(I)	19.71 (1.66)	7.92 (0.40)
Wilson B-factor	58.65	51.9
R-merge	0.1324 (2.489)	0.1599 (4.302)
R-meas	0.1359 (2.553)	0.1707 (4.588)
R-pim	0.0306 (0.5623)	0.05921 (1.576)
CC1/2	1 (0.589)	0.997 (0.0741)
CC*	1 (0.861)	0.999 (0.372)
Reflections used in refinement	29423 (2907)	29151 (2900)
Reflections used for R-free	1494 (139)	1483 (141)
R-work	0.2064 (0.3019)	0.2118 (0.3811)
R-free	0.2555 (0.3081)	0.2501 (0.3900)
CC(work)	0.970 (0.679)	0.950 (0.611)
CC(free)	0.950 (0.645)	0.934 (0.614)
Number of non-hydrogen atoms	5058	5215
macromolecules	5020	5083
solvent	38	132
Protein residues	633	639
RMS(bonds)	0.008	0.007
RMS(angles)	1.02	0.93
Ramachandran favored (%)	94.90	96.82
Ramachandran allowed (%)	4.63	3.02
Ramachandran outliers (%)	0.48	0.16
Rotamer outliers (%)	0.35	0.69
Clashscore	17.62	12.65
Average B-factor	81.29	68.15
macromolecules	81.42	68.46
solvent	63.76	56.18
